# Humic Acid Modulates Ionic Homeostasis, Osmolytes Content, and Antioxidant Defense to Improve Salt Tolerance in Rice

**DOI:** 10.3390/plants12091834

**Published:** 2023-04-29

**Authors:** Mohamed Abu-Ria, Wafaa Shukry, Samy Abo-Hamed, Mohammed Albaqami, Lolwah Almuqadam, Farag Ibraheem

**Affiliations:** 1 Botany Department, Faculty of Science, Mansoura University, Mansoura 35516, Egypt; 2 Botany and Microbiology Department, College of Science, King Saud University, Riyadh 11451, Saudi Arabia; 3 Biology Department, College of Science, Imam Abdul Rahman Bin Faisal University, Damam 31441, Saudi Arabia; 4 Biology and Chemistry Department, Al-Qunfodah University College, Umm Al-Qura University, Al-Qunfodah 21912, Saudi Arabia

**Keywords:** rice, salinity, humic acid priming, antioxidants, osmolytes

## Abstract

The sensitivity of rice plants to salinity is a major challenge for rice growth and productivity in the salt-affected lands. Priming rice seeds in biostimulants with stress-alleviating potential is an effective strategy to improve salinity tolerance in rice. However, the mechanisms of action of these compounds are not fully understood. Herein, the impact of priming rice seeds (cv. Giza 179) with 100 mg/L of humic acid on growth and its underlaying physiological processes under increased magnitudes of salinity (EC = 0.55, 3.40, 6.77, 8.00 mS/cm) during the critical reproductive stage was investigated. Our results indicated that salinity significantly reduced Giza 179 growth indices, which were associated with the accumulation of toxic levels of Na^+^ in shoots and roots, a reduction in the K^+^ and K^+^/Na^+^ ratio in shoots and roots, induced buildup of malondialdehyde, electrolyte leakage, and an accumulation of total soluble sugars, sucrose, proline, and enzymic and non-enzymic antioxidants. Humic acid application significantly increased growth of the Giza 179 plants under non-saline conditions. It also substantially enhanced growth of the salinity-stressed Giza 179 plants even at 8.00 mS/cm. Such humic acid ameliorating effects were associated with maintaining ionic homeostasis, appropriate osmolytes content, and an efficient antioxidant defense system. Our results highlight the potential role of humic acid in enhancing salt tolerance in Giza 179.

## 1. Introduction

Rice (*Oryza sativa* L.) is an essential stable crop worldwide. It grows in many parts of the world, and it is used as a principal food for more than 50% of the people of the world [1]. Rice provides 50–80% of daily calories with a high nutritional value of carbohydrates, fats, proteins, vitamins, and minerals. It also contains many bioactive phytochemicals with anticancer, antioxidant, anti-inflammation, and antidiabetic activities [2,3]. 

Rice is categorized as a salinity susceptible cereal and loses 12% of its yield for every 1 mS/cm rise in electrical conductivity above its salt sensitivity threshold (3 mS/cm) [4]. Therefore, salinity stress is a major constraint for rice production worldwide. Unfortunately, about half of the world’s irrigated lands are projected to suffer from salinity stress by 2050 [5]. Salinity-stressed plants suffer osmotic stress during the early stages of salt stress and experience ionic stress under long-term stress [6]. Osmotic stress induces physiological dysfunctions that result in the reduction in the efficiency of various critical processes including water uptake, cell expansion, cell division, leaf area, photosynthesis, and overall plant growth [7,8,9]. Ionic stress disrupts cellular functions and ionic homeostasis and elicits production of reactive oxygen species (ROS) that can harm proteins and cell membranes [10]. Na^+^ is the most frequent harmful ion, causing direct cellular damage to plants and antagonistically inhibits K^+^ uptake [11]. Excess levels of Na^+^ also interfere with a variety of biological processes such as photosynthesis, enzyme activity, and protein synthesis. Therefore, maintaining a lower cytosolic Na^+^ and a perfect K^+^/Na^+^ ratio is essential for sustaining rice development under salinity stress [12].

Seed priming is one of the important techniques that improve the plant resistance against future exposure to abiotic stresses like salinity [13]. For instance, humic acid can be utilized to enhance plant growth, mineral nutrient uptake, and salinity stress tolerance [14]. Priming plant seeds with humic acid positively modulates the hormonal-signaling pathways and several functional/regulatory stress-responsive genes [15]. Humic acid enhances plant growth and physiology through increasing the intensity of photosynthesis, hormonal activity, cell membrane permeability, and nutrient uptake under abiotic stresses [16,17]. It also activates the antioxidant defense system and, thus, reduces the levels of ROS and regulates the expression of genes encoding aquaporins in the tonoplast [18].

Rice breeders have identified Giza 179 as a high-yielding cultivar under the Egyptian non-saline agricultural growth conditions; it also shows a considerable level of tolerance against salinity in salt-affected lands [19]. To our knowledge, the possible improvement of Giza 179 growth and physiological responses during the critical reproductive stage under non-saline and saline conditions by humic acid priming has not been investigated. Therefore, the aim of the current study was to assess the impact of humic acid on the growth and its underlaying physiological processes, if any, in Giza 179 plants at the reproductive stage under non-saline and saline conditions. Our hypothesis is that, as a biostimulant and stress alleviator, humic acid can improve Giza 179 growth under non-saline conditions and mitigate the salinity-induced responses under saline water irrigation by maintaining an efficient interplay among ionic homeostasis, active osmolyte production, and the antioxidant defence system. Therefore, the specific objectives of the current investigation are (1) To evaluate the possible enhancing effects of humic acid, if any, on the growth of Giza 179 under non-saline, as well as under increased levels of salinity in saline water irrigation; and (2) Identify the possible physiological mechanisms underlaying humic acid-induced effects on Giza 179 growth. This was carried out by priming Giza 179 seeds in 100 mg humic acid/l before planting. In addition, salinity stress was initiated by irrigation with increased levels of seawater (tap water, 5%, 10%, and 12.5%, which correspond to EC values of 0.55, 3.40, 6.77, and 8.00 mS/cm). At the reproductive stage, growth performance, mineral homeostasis, osmo-protection, oxidative stress markers, and antioxidant properties in rice plants under saline and non-saline conditions were monitored.

## 2. Results

### 2.1. Effect of Humic Acid Priming on Plant Growth under Non-Saline and Saline Conditions

Salt-stressed Giza 179 plants showed apparent growth retardation in response to increasing levels of salinity stress (5%, 10%, and 12.5% seawater). Compared to the control plants, the severe salt stress (12.5% seawater) showed a maximum reduction of 26.8%, 73.4%, 75.0%, and 21.5% in plant height, fresh weight, dry mass, and leaf area, respectively (Table 1). Compared to rice plants that were not treated with humic acid, humic acid-treated rice plants had better growth in both non-saline and saline conditions (Table 1). Under non-saline conditions, humic acid-treated Giza 179 rice plants showed 8.6%, 19.8%, 28.9%, and 25.2% higher plant height, fresh weight, dry mass, and leaf area, respectively, than untreated rice plants. Humic acid alleviated the salinity-induced retardation in all growth parameters at all tested salinity levels. For instance, at 5%, 10%, and 12.5%, the percentage of growth enhancement in humic acid-treated plants was 9.0%, 9.0%, and 5.8% in plant height and 25%, 30.1%, and 72.6% in plant fresh weight seawater, respectively, compared to humic acid non-treated rice plants. The corresponding percentages of increase were 24.9%, 43.3%, and 67.7% in plant dry weight and 11.7%, 7.8%, and 5.2%, in leaf area, respectively (Table 1).

### 2.2. Effect of Humic Acid Priming on K^+^ and Na^+^ Concentration

Salinity stress significantly increased the content of Na^+^ in shoots and roots (Table 2). Compared to the control plants, the salinity-induced increases in shoots’ Na^+^ content approached 27.8%, 51.4%, and 72.2% at 5%, 10%, and 12.5% seawater, respectively, whereas the corresponding percentages of increase in roots’ Na^+^ content were 17.7%, 32.9%, and 46.8%, respectively. In contrast to Na^+^ responses, salinity stress decreased K^+^ content and the K^+^/Na^+^ ratio in shoots and roots. Compared to the control plants, the maximum salinity-induced reductions in K^+^ content and the K^+^/Na^+^ ratio in shoots were 12.1% and 49.0%, whereas the corresponding records in roots were 36.8% and 56.7% at 12.5% seawater, respectively.

Interestingly, in salinity-stressed plants, seed priming with humic acid significantly decreased shoots’ Na^+^ content while significantly increasing roots’ Na^+^ content in comparison with those in shoots and roots of humic acid non-treated stressed plants (Table 2). In addition, seed priming with humic acid significantly decreased shoots’ K^+^ content but increased roots’ K^+^ content. Further, humic acid application increased shoots’ and roots’ K^+^/Na^+^ ratio under non-saline and saline conditions. In salinity-non-stressed plants, the humic acid-induced increase in the K^+^/Na^+^ ratio was 20.3% in shoots and 0.5% in roots compared to the control plants. In salinity-stressed plants, the humic acid-induced increase in shoots’ K^+^/Na^+^ ratio was 10.3%, 5.4%, and 2.3% at 5%, 10%, and 12.5% seawater, respectively. The corresponding roots’ K^+^/Na^+^ ratio was 7.6%, 17.1%, and 31.7%, respectively, compared to humic acid non-treated stressed plant.

### 2.3. Effect of Humic Acid Priming on Salinity-Induced Oxidative Damages

**Figure 1:**
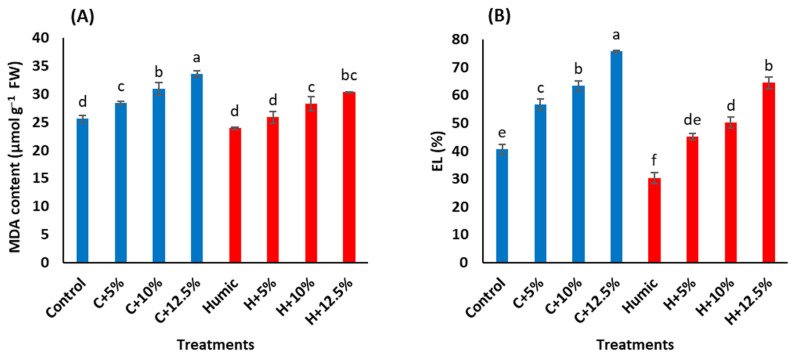
Effect of seed priming with humic acid (100 mg/L) on oxidative stress parameters (**A**) MDA content and (**B**) EL percentage of Giza 179 plants grown under increased levels of seawater. Values are the means ± SE of three replicates. Different bar letters indicate significant differences at *p* < 0.05.

In rice shoots, increased levels of salinity increased malondialdehyde (MDA) content and electrolyte leakage (EL) relative to the control treatments (Table 3). Under non-saline conditions, seed priming with humic acid had no obvious effect on MDA content, while it significantly decreased EL. Under saline conditions, MDA content and EL were significantly decreased in humic acid-treated plants, compared to humic acid non-treated plants. The humic acid-induced reduction in MDA level approached 9.1%, 8.7%, and 9.7% at 5%, 10%, and 12.5% seawater, whereas the corresponding levels of such reduction in EL were 20.2%, 20.6%, and 14.9%, respectively, relative to their levels in shoots of humic acid non-treated Giza 179 plants (Figure 1A,B).

### 2.4. Effect of Humic Acid Priming on Osmolytes Content

**Figure 2:**
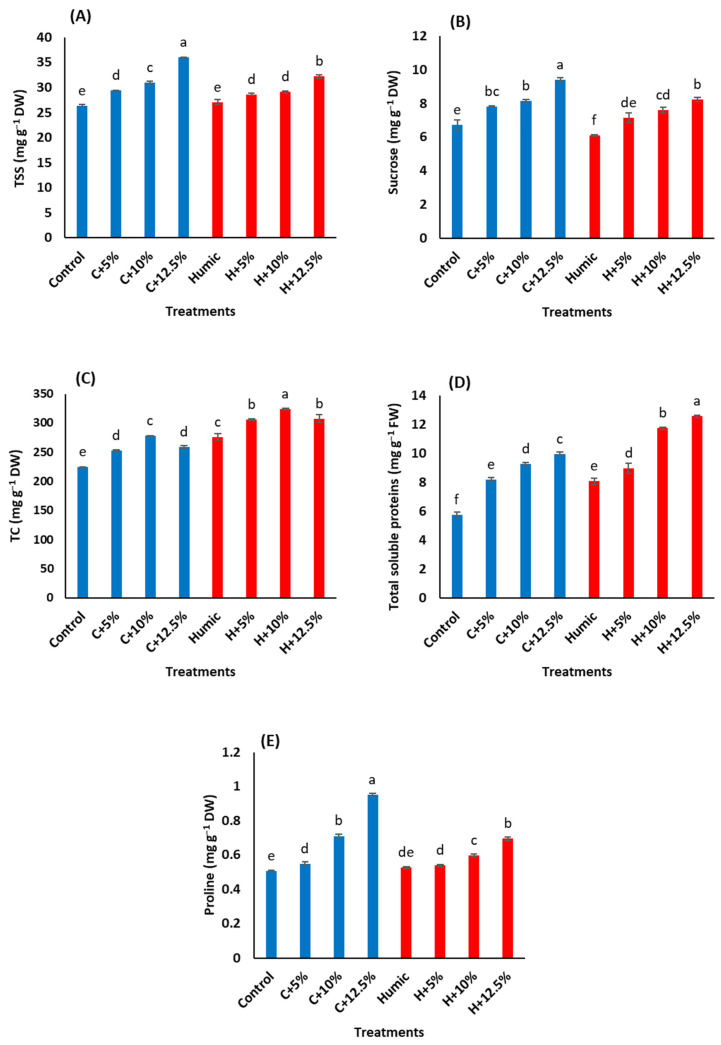
Effect of seed priming with humic acid (100 mg/L) on osmolyte content (**A**) total soluble sugars, (**B**) sucrose, (**C**) total carbohydrates, (**D**) soluble proteins, and (**E**) proline content of Giza 179 plants grown under increased levels of seawater. Values are the means ± SE of three replicates. Different bar letters indicate significant differences at *p* < 0.05.

In general, salinity stress increased TSS, sucrose, TC, soluble proteins, and proline contents in Giza 179 shoots in comparison with the control plants (Figure 2 and Table 3). The extent of salinity-induced accumulation in these osmolytes generally increased as the salinity level increased. The highest salinity level (12.5% seawater) resulted in a maximum increase in TSS (36.8%, Figure 2A), sucrose (39.9%, Figure 2B), total soluble proteins (73.6%, Figure 2D), and proline (88.3%, Figure 2E)) contents compared to the control plants. In salinity-stressed plants, priming with humic acid generally decreased TSS, sucrose, and proline contents in comparison with those of humic acid non-treated stressed plants. In contrast, humic acid priming induced an accumulation of TC (Figure 2C) and total soluble proteins in the control and salinity-stressed plants (Table 3). Compared to humic acid non-treated Giza 179 plants, the humic acid-induced accumulation of TC approached 22.7%, 21.2%, 16.6%, and 18.9% at 0%, 5%, 10%, and 12.5% seawater, respectively, whereas the corresponding values in total soluble proteins were 40.7%, 9.2%, 26.7%, and 26.2%, respectively.

### 2.5. Effect of Humic Acid Priming on Antioxidant System

**Figure 3:**
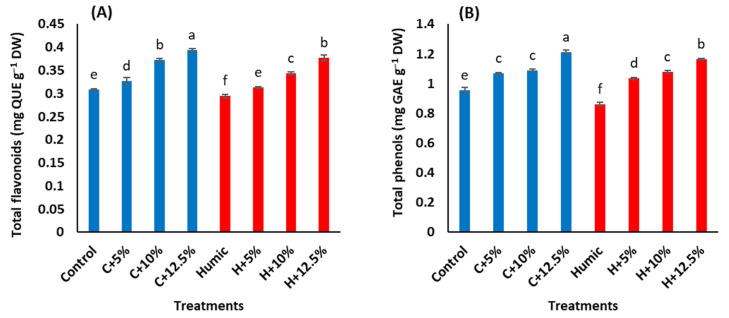

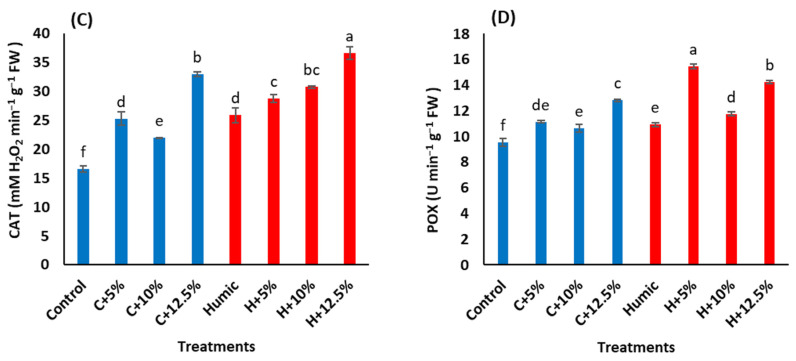
Effect of seed priming with humic acid (100 mg/L) on antioxidant activities, (**A**) total flavonoids, (**B**) total phenols, (**C**) catalase activity (CAT), and (**D**) peroxidase activity (POX) of Giza 179 plants grown under increased levels of seawater. Values are the means ± SE of three replicates. Different bar letters indicate significant differences at *p* < 0.05.

The interactive effects of salinity stress and priming with humic acid on the flavonoids and total phenols in the leaves of Giza 179 plants are shown in (Figure 3A,B). Compared to the control plants, increasing salinity levels caused a significant increase in flavonoids and total phenols with a maximum increase at 12.5% seawater. Seed priming with humic acid significantly decreased the contents of flavonoids and total phenols in leaves of Giza 179 plants under non-saline and saline conditions (Table 3). In salinity-non stressed plants, humic acid priming induced a decrease of 4.5% in flavonoids and 9.7% in total phenols. In salinity-stressed plants, the humic acid-induced decreases in flavonoids and total phenols were 4.5% and 3.2% at 5%, 7.5%, and 1% at 10% and 4.2%, and 4% at 12.5% seawater, respectively, in comparison with those in humic acid non-treated stressed plants.

Regarding antioxidant enzyme activities, the increased salinity stress levels triggered a significant increase in the activities of both CAT and POX (Table 3). Salinity-induced increases in CAT activities were 52.7%, 32.4%, and 98.9% at 5%, 10%, and 12.5% seawater, whereas in POX, such increases were 16.8%, 11.7%, and 34.2%, respectively (Figure 3C,D). In salinity-non stressed plants, seed priming with humic acid increased the activities of CAT by 56.2% and POX by 14.3%. In salinity-stressed plants, humic acid priming induced increases in CAT activities by 13.7%, 40.2%, and 11.3% at 5%, 10%, and 12.5% seawater, whereas the corresponding increases in POX activity were 38.7%, 10.2%, and 11.1%, respectively, in comparison with humic acid non-treated stressed Giza 179 plants.

### 2.6. Principal Component Analysis and Pearson Correlations among Treatments

**Figure 4:**
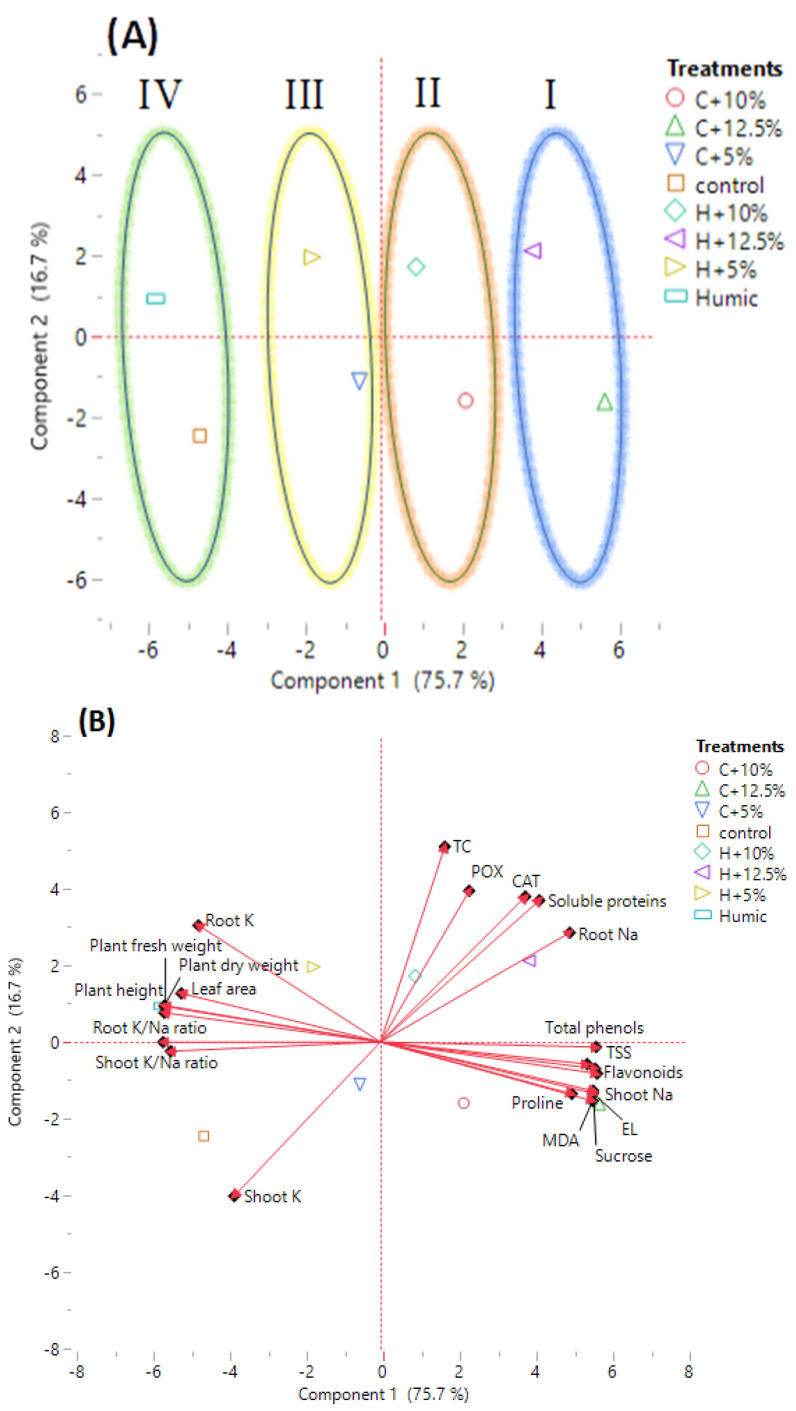
Principal component analysis (PCA) of growth, physiological, biochemical, and mineral traits in the investigated humic acid (100 mg/L) treated and non-treated Giza 179 plants grown under increased levels of seawater. (**A**) PCA score plot and (**B**) PCA biplot of 21 measured traits.

The tested variables, including growth indices (plant height, plant fresh and dry weight, and leaf area), mineral content in shoots and roots (Na^+^, K^+^, and K^+^/Na^+^ ratio), oxidative stress markers (EL and MDA), osmolyte content (TSS, sucrose, TC, soluble proteins, and proline), non-enzymatic antioxidants (total phenols and flavonoids), and antioxidant enzymes (CAT and POX) were subjected to a principal component analysis (PCA) (Figure 4).

The larger percentage of variance was described by the first principal component (PC1), which contributed for 75.7% of the variance among the tested parameters. The smaller percentage of variance (16.7%) was described by the second principal component (PC2). PC1 showed the variance between non-saline and saline conditions, while PC2 showed the variance between humic acid-treated and non-treated rice plants under non-saline and saline conditions. A PCA score plot separated humic acid-treated and non-treated Giza 179 plants growing under saline and non-saline conditions into four main groups. Group 1 included severe salt-stressed Giza 179 plants, treated and non-treated with humic acid (H + 12.5% and C + 12.5% seawater, respectively). Group 2 included moderate salt-stressed Giza 179 plants, treated and non-treated with humic acid (H + 10% and C + 10% seawater, respectively). Group 3 included low salt-stressed Giza 179 plants treated and non-treated with humic acid (H + 5% and C + 5% seawater, respectively), and Group 4 included humic acid-treated and non-treated Giza 179 plants grown under normal conditions (Figure 4A).

Shoots Na^+^, oxidative stress markers (EL percentage and MDA content), Osmolytes (TSS, sucrose, and proline contents), and non-enzymic antioxidants (total phenols and flavonoids) were strongly associated with moderate and high salinity levels (C + 10% and C + 12.5% seawater), whereas roots Na^+^, osmolytes (TC and total soluble protein), and antioxidant enzymes (CAT and POX) were strongly associated with the humic acid-treated Giza 179 plants under moderate and high salinity (H + 10% and H + 12.5% seawater). On the other hand, growth parameters (plant height, plant fresh and dry weight, and leaf area), roots K^+^, and shoots’ and roots’ K^+^/Na^+^ ratio were strongly associated with the humic acid-treated Giza 179 plants under normal and low salinity levels (Humic and H + 5% seawater), whereas shoots K^+^ appeared strongly associated with plants not treated with humic acid under normal and low salinity levels (Control and C + 5% seawater) (Figure 4B).

**Figure 5:**
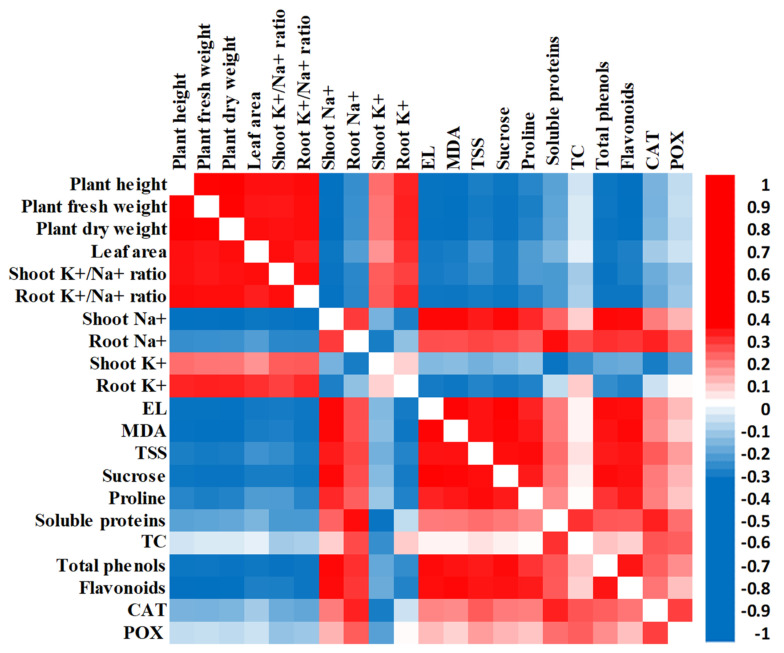
Heatmap of Pearson correlation analysis of all investigated traits in humic acid (100 mg/L) treated and non-treated Giza 179 plants grown under increased levels of seawater. Red and blue colors represent positive and negative correlations, respectively, according to the color scale.

A heatmap correlation analysis showed a substantial positive relationship between growth parameters (plant height, plant fresh weight, plant dry weight, and leaf area), and shoots’ and roots’ K^+^ and K^+^/Na^+^ ratio. However, a negative relationship was observed between growth parameters and the rest of the investigated variables that showed a positive relationship with each other (Figure 5).

## 3. Discussion

### 3.1. Humic Acid Priming Improves Giza 179 Plant Growth under Non-Saline and Saline Conditions

Salinity is one of the deleterious abiotic environmental stresses that creates ionic, osmotic, and oxidative stresses that induce significant metabolic alterations, decrease water and nutrient uptake, and subsequently reduce plant growth and development [20,21]. In the present study, salinity stress levels displayed profound negative effects on the plant height, fresh and dry weights, and leaf area of Giza 179 plants in a dose-dependent manner (Table 1). Our results are consistent with the previous reported reduction in rice growth under salinity stress [22,23]. The retardation in growth of the salt-stressed Giza 179 plants is attributed to (1) The salinity-induced osmotic, ionic, and oxidative stresses [24]; (2) The reduced chlorophyll content and its associated reduction in the level of photosynthates, which decreases the activity of meristem cells; and (3) The disturbance in water balance that reduces cell elongation and cell division [7,25]. These results are consistent with those from a heatmap correlation analysis, which showed a large negative relationship between growth parameters and shoots’ and roots’ Na^+^ content and oxidative stress markers (EL percentage and MDA content) (Figure 5).

Seed priming with humic acid increased Giza 179 growth under non-saline conditions and alleviated the adverse effects of salinity on its growth (Table 1). These findings give insights into the beneficial effects of humic acid on Giza 179 plant growth under non-saline and saline conditions. Similar growth-promoting effects of humic acid have been reported in other rice cultivars [26,27], soybean seedlings [28], and maize [29]. These findings are supported by the results of PCA analysis (Figure 4A) that revealed differences between humic acid-treated and non-treated rice plants growing in non-saline and saline conditions. The general promotive effects of humic acid on plant growth are due to its role in the induction of several biochemical changes in membranes and cytoplasmic components once it enters plant cells, resulting in enhanced plant growth and salinity stress tolerance [30]. Such humic acid growth-promoting effects are attributed to its auxin-like effects, as well as its ability to increase cell membrane permeability, root uptake capacity for water and nutrients, and hormonal activity [31,32,33]. It is worth mentioning that humic acid enhanced Giza 179 germination and post-germination growth via the induction of gibberellins (GA_3_) and α-amylase activity, particularly at 3 days after priming, which is the stage at which intensive metabolic conversion is required to support the growth of the germinating seeds (not shown). Such responses significantly shorten the time interval between radical/plumule emergence and seedling establishment and, thus, protect Giza 179 seedlings against salinity stress during the critical phase of seedling establishment, which coincides with the mechanism of action of various seed priming biostimulants in improving germination and post-germination growth [34]. In fact, humic acid has been suggested as a chemical eustress that stimulates the growth and development of plants [35,36]. These findings suggest that seed priming with humic acid induces a significant alteration in the physiological properties of Giza 179 plants growing in non-saline and saline conditions.

### 3.2. Humic Acid Priming Alleviates Salinity-Induced Stress via Regulation of the Ionic Content

The accumulation of Na^+^ and Cl^-^ ions in plant tissues is the most damaging result of salt stress since they disrupt ion homeostasis, cause physiological disorders, and reduce the uptake of K^+^ that is essential for plant growth and development [37]. Salinity stress reduces the K^+^/Na^+^ ratio because of high Na^+^ influx, which induces K^+^ ion efflux [38]. In the current study, salt stress increased Na^+^ levels in shoots and roots of Giza 179 plants but decreased K^+^ and the K^+^/Na^+^ ratio. These responses induce high ion toxicity and eventual growth retardation (Table 2). Our results are consistent with the reported alteration in ion homeostasis in rice shoots and roots, in response to salinity, by increasing Na^+^ accumulation while decreasing K^+^ and the K^+^/Na^+^ ratio in both organs [24,39]. In fact, salinity tolerance in rice was achieved through decreasing Na^+^ root-to-shoot distribution and an increase of Na^+^ accumulation in rice roots by the unloading of Na^+^ from xylem [40,41]. In addition, salt tolerant rice cultivars diminished Na^+^ accumulation in their leaves by controlling the expression of OsHKT1;5, which mediates Na^+^ relocation from xylem to xylem-parenchyma cells [42]. According to Chakraborty et al. [43], excess Na^+^ in the cytoplasm damages plant cell membranes and organelles, affects K^+^ nutrition, and reduces plant physiological systems such as photosynthesis, cytosolic enzymes, and metabolism.

Humic acid priming decreased the level of the shoots’ Na^+^, while it increased the roots’ Na^+^ accumulation (Table 2). This effect was attributed to the role of humic acid in enhancing the reallocation of Na^+^ to xylem parenchyma cells and reducing the net flow of Na^+^ into the shoot via controlling the expression of HKT1 that encodes sodium influx transporter in plasma membrane of xylem parenchyma cells. Such a response partially explains the growth promotive effects of humic acid under salt stress [44]. Despite the roots’ Na^+^ level in humic acid-treated plants being higher than those in non-treated plants, the roots’ K^+^/Na^+^ ratio was maintained and increased in humic acid-treated rice plants (Table 2). PCA biplot showed that roots’ and shoots’ K^+^/Na^+^ ratios were mostly associated with humic acid-treated plants under non-saline conditions. Meanwhile, roots’ Na^+^ and shoots’ Na^+^ were mostly associated with humic acid-treated and non-treated Giza 179 plants grown under severe salt stress (H + 12.5% and C + 12.5% seawater), respectively (Figure 4B).

### 3.3. Humic Acid Priming Alleviates Salinity-Induced Oxidative Damage

The salinity-induced accumulation of Na^+^ inside plant cells induces the production of ROS, which induces lipid peroxidation and damages cellular membranes leading to leakage of cellular electrolytes [42,45]. In the present study, Giza 179 plants are exposed to salinity-increased EL percentages and MDA content in their leaves (Figure 1A,B). MDA is a by-product of polyunsaturated fatty acid oxidation that is commonly employed as a marker of lipid peroxidation and cell membrane damage in response to abiotic stimuli [46,47]. These results agree with the reported oxidative stress and its associated lipid peroxidation, increased membrane permeability, and outflows of the electrolytes in salinity-stressed rice plants [48,49]. Interestingly, the heatmap correlation analysis showed a substantial positive association between shoots’ Na^+^, EL percentage, and MDA content (Figure 5).

Seed priming with humic acid decreased the EL percentage and MDA content in salt-stressed Giza 179 plants (Figure 1A,B), indicating that humic acid successfully alleviated the salinity-induced oxidative damage. Similar effects of humic acid on lowering EL have been reported in salinity-stressed bean plants [32] and almond rootstocks [50]. In addition, humic acid reduced the salt-induced lipid peroxidation and oxidative stress in *Urochondra setulosa* [51]. Such effects of humic acid are most likely linked to its ability in decreasing shoots’ Na^+^ content (Table 2) and, thus, it maintains membrane integrity as an important determinant of plant stress tolerance [23]. The PCA biplot supports this hypothesis and revealed a positive relationship between shoots’ Na^+^, MDA content, and EL percentage with severe salt stress (C+12.5% seawater) (Figure 4B).

### 3.4. Humic Acid Priming Modulates Salinity-Induced Disruption in Osmolyte Content

Osmotic adjustment is a useful strategy for tolerating salt-induced osmotic stress where the compatible solutes lower the cell osmotic potential and scavenge excess ROS [52,53]. In the current study, salinity stress significantly increased TSS, sucrose, TC, proline, and soluble proteins (Figure 2A–E). These findings agree with the reported accumulation of TSS and sucrose in mature leaves of salinity-stressed cv. Khao Dawk Mali 105 rice seedlings [54]. Likewise, a similar accumulation of proline and soluble proteins has been reported in salinity-stressed plants [22,24,55]. Under salinity stress, high sugar content contributes to osmotic adjustment, diminishes Na^+^ inhibitory effects, removes excess ROS, and maintains protein and membrane in rice plants [22] and olive seedlings [56]. Likewise, proline acts as an osmo-protectant, which contributes to osmotic stress tolerance [57,58]. In addition, the role of soluble proteins in maintaining the osmotic adjustment, increasing membrane stability, and activating antioxidant defense mechanisms in response to salinity stress has been reported [55]. Yan et al. [41] indicated that salinity stress induced an increase in rice root soluble proteins, which contributed to decreasing root sap osmotic potential and led to more water uptake, thus overcoming the toxic effects of salinity stress. This agreed with [59], which reported the accumulation of hydrophilic proteins from late embryogenesis-abundant protein superfamilies in plant cells that suffer osmotic stress where these soluble proteins protect higher plants from osmotic stress damage caused by salinity. One of the main salt-tolerant mechanisms in plants is the synthesis of stress proteins that reduce salinity-induced osmotic stress [38].

Seed priming with humic acid decreased the salinity-elicited increment in TSS, sucrose, and proline while it increased TC and soluble proteins. The mechanism underlying this reduction is probably due to the ability of humic acid to maintain active growth under salinity. This was supported by the higher TC content in humic acid-treated Giza 179 plants than non-treated plants, reflecting the availability of carbon skeleton and energy resources for maintaining the active growth under salinity. It has been reported that the accumulation of soluble sugars in rice can reduce growth under salinity; this was probably due to the reduced utilization of starch in actively growing tissues [60]. They also indicated that the rice plants that convert sugar to starch and maintain higher starch accumulation under salinity possess a larger biomass, higher number of leaves, and maintain photosynthesis by minimizing the inhibition by sugars. Consistently, [61] revealed that a higher concentration of sugars in cytoplasm could inhibit carbon metabolism and cause sugar injury. Therefore, partitioning sugars into starch may help to avoid metabolic alteration through lowering the inhibition effect caused by excess amounts of sugars in cytoplasm.

Priming Giza 179 rice seeds with humic acid decreased the salinity-induced increase in proline. Similar effects of humic acid have been reported in barley [62], pepper [63], and maize [29]. The decrease in proline content was explained by [64], which stated that a high concentration of compatible solutes, such as proline, may have deleterious effects on plant growth and metabolism. On the other hand, the humic acid-induced protein synthesis in salinity-stressed Giza 179 plants in the current study agrees with the reported induction of leaf soluble protein in salinity-stressed almond rootstocks after the application of humic acid [50]. The role of humic acid in increasing protein biosynthesis comes from its role in increasing photosynthesis, mineral nutrient uptake, transcriptional activation, and protein synthesis [31,65]. The correlation analysis by PCA confirmed these explanations as growth parameters, TC, and the soluble proteins being mostly associated with humic acid-treated plants whereas TSS, sucrose, and proline were mostly associated with plants that were not treated with humic acid (Figure 4B).

### 3.5. Humic Acid Priming Modulates Salinity-Induced Changes in Antioxidant System

Salt stress induces stomatal closure that reduces CO_2_ availability and fixation in the leaf tissues and decreases the consumption of reducing powers that can trigger ROS generation, such as hydrogen peroxide, superoxide, singlet oxygen, and hydroxyl radicals. These events culminate in an oxidative stress [9,66,67]. As a result, salinity-stressed plants activate their enzymatic and non-enzymatic antioxidant defence mechanisms that work together to scavenge ROS and, thus, protect themselves from oxidative stress [68]. In the present study, salinity-stressed Giza 179 significantly increased the activities of the antioxidant enzymes (CAT & POX), as well as the level of the non-enzymic antioxidants (total phenols and flavonoids) in their leaves compared to the control plants (Figure 3A–D). The increases in CAT and POX activities in Giza 179 are in line with the reported ability of rice [69,70] and maize plants [29] to boost CAT and POX activities in response to salinity to mitigate the damaging consequences of salt stress. Such higher antioxidant enzyme activities in salinity-stressed rice plants were correlated with a reduced level of ROS generation [71]. Similarly, the increase in the phenols and flavonoids antioxidants in Giza 179 are consistent with the reported increase of these compounds in salinity-stressed Nonabokra, Swarna, OM4900, and BC15TB rice cultivars [45,72]. In fact, the induction of these compounds has been suggested as an adaptive mechanism of rice under salt stress [72] due to their efficiency in scavenging ROS, which is attributed to their high reactivity as electron donors to stabilize the unpaired electron. Furthermore, [73,74] explained the rise in salinity-induced flavonoids by suggesting that flavonoid-related key genes may be involved in reducing salt-induced oxidative damage. Our heatmap correlation analysis showed a strong positive association among oxidative stress markers (MDA and EL) and non-enzymatic (total phenols and flavonoids) antioxidants (Figure 5).

Seed priming with humic acid significantly increased the activities of the antioxidant enzymes (CAT and POX) (Figure 3C,D). However, it decreased the content of non-enzymatic antioxidants (total phenols and flavonoids) (Figure 3A,B) in the leaves under non-saline and saline conditions. The humic acid application to rice root increased both the ROS production and the activities of antioxidant enzymes, and such increases in ROS were not associated with a negative effect on plants but rather with the beneficial effect of increasing root growth [75]. In addition, humic acid enhanced CAT activity and the production of ROS in maize plants [76]. The increase in ROS to a certain threshold may be a prerequisite cue for induction of the antioxidant enzymes and mediation of the plant growth [76,77]. The humic acid-induced activities of CAT and POX in Giza 179 most likely contribute to its improved growth under salinity stress. Similar humic acid protective effects against salinity stress via induction of CAT and POX enzyme activities have been reported in wheat [78]. On the other hand, the humic acid-induced reduction in non-enzymic antioxidants in Giza 179 (Figure 3A,B) has also been reported in leaf total phenolic content in salinity-stressed *Vitex trifolia* plant [79]. In addition, the reduction of total phenolics content in shoots and roots of arsenic-stressed rice plants in response to rice seed priming with K-humate has been reported [80]. The reduction in the content of such secondary metabolites may be attributed to the ability of humic acid to maintain active growth as evidenced by humic acid-treated plants’ growth metrics (Table 1), which shunts resources away from the biosynthesis of secondary metabolites [81]. PCA analysis confirmed these results as growth parameters and antioxidant enzymes (CAT & POX) were associated with humic acid-treated Giza 179 plants, whereas non-enzymatic (total phenols and flavonoids) antioxidant was associated with plants that were not treated with humic acid (Figure 4B).

## 4. Materials and Methods

The seeds of Egyptian rice cultivar cv. “Giza 179” were obtained from Rice Research and Training Center, Sakha, Kafr El-Sheik, Egypt. Humic acid as stress alleviator was purchased from Sigma–Aldrich company (CAT No. 53680, St. Louis, MO, USA) and the seawater was obtained from Gamasa, Mediterranean Sea, Egypt.

### 4.1. Experimental Setup, Treatments, and Tissue Collection

Rice seeds were surface sterilized using sodium hypochlorite (3.6%) for 15 min, washed thrice with sterilized distilled water, and the washed seeds were allocated into two groups. The first group was soaked in distilled water (control), whereas the second one was soaked in 100 mg/L humic acid with (pH 7) in the dark at 27 ± 2 °C for 72 h. The two seed groups were sown in 10 pots (five pots each). Each pot was 25 cm in diameter and contained 7 kg soil, and it had 65 uniform size and healthy seeds. Soil chemical characteristics were determined in 1:2.5 soil extracts and were EC 1.53 mS/cm, pH 8.35, organic matter 2.34%, C/N ratio 5.44, cations in meq/100 g soil (Na^+^ 2.18, K^+^ 0.20, Ca^2+^ 0.94, Mg^2+^ 0.65) and anions in meq/100 g soil (HCO_3_^−^ 0.58, Cl^−^ 1.87, SO_4_^2−^ 1.57). After 28 days of sowing, uniform rice plantlets were transplanted (15 plantlet/pot) into 40 larger pots (20 pots for each group, 30 cm in diameter) containing 10 kg of the same soil with drainage holes at the bottom to facilitate discharge of the excess seawater after irrigation with seawater and, thus, minimize salt accumulation in the soil. The transplanted plantlets were maintained in the greenhouse until complete recovery and establishment. Pots with homogeneous-recovered plantlets were thinned to 10 plants/pot and used for application of the salinity stress, which was imposed by application of seawater dilutions of 0%, 5%, 10%, and 12.5% with EC values of 0.55, 3.40, 6.77, and 8.00 mS/cm, respectively. The stock seawater has EC of 54.6 mS/cm, pH 10.5, cations in ppm (Na^+^ 7400, K^+^ 340, Ca^2+^ 519, Mg^2+^ 1395) and anions in ppm (HCO_3_^−^ 91, Cl^−^ 1786).

A factorial experiment was arranged in a complete randomized design with two main factors: (1) Amendment with two levels: control and 100 mg/L humic acid; and (2) Salinity stress with four levels: 0%, 5%, 10%, and 12.5% seawater. Pots with the established controls and humic acid-treated Giza 179 plants were allocated into four subsets with a total of eight treatments as follow: (1) Control (C); irrigated with tap water; (2) C + 5%; irrigated with 5% seawater dilution; (3) C + 10%; irrigated with 10% seawater dilution; (4) C + 12.5%; irrigated with 12.5% seawater dilution; (5) Humic (H); humic acid pre-treated plants irrigated with tap water, (6) H + 5%; humic acid pre-treated plants irrigated with 5% seawater dilution; (7) H + 10%; humic acid pre-treated plants irrigated with 10% seawater dilution; and (8) H + 12.5%; humic acid pre-treated plants irrigated with 12.5% seawater dilution. Both the control and humic acid-treated plants were irrigated with equal volumes of either tap water or each of the corresponding seawater dilution every 4 days.

Seventy-five days after transplantation, plants were collected from each treatment, washed twice with distilled water, blotted dry, and separated into roots and shoots. Tissues collected from each treatment were separated into two subsets; the first subset was frozen instantly in liquid N and transferred to –80 °C and used for MDA and enzyme analysis, whereas the second subset was used for measuring growth parameters (fresh weight, shoot and root lengths, leaf area) and then dried to a constant weight at 70 °C in an electric oven. The dried tissues were then crushed into uniform powder using a stainless-steel grinder and used for elemental and biochemical analyses.

### 4.2. Plant Growth Parameters

A caliber was used to measure root and shoot lengths, whereas fresh plants and dry weights were measured using a digital balance. Leaf area was measured according to Palaniswamy et al. [82] using this equation: leaf area (cm^2^) = length × width × 0.75.

### 4.3. Ion Contents

Known weights of the powdered shoot and root samples were digested in a mixture of 5 mL nitric acid and 1 ml of perchloric acid as described previously [83]. Flame photometer (PFP7, Jenway) was used to measure the content of Na^+^ and K^+^. Data were expressed as mmol g^−1^ DW.

### 4.4. Determination of Osmolytes Content

The content of osmolytes, including TSS, sucrose, TC, soluble proteins, and proline, were analyzed spectrophotometrically using a Shimadzu spectrophotometer (model UV-160A). TSS and sucrose were extracted using the method of [84] by incubating 0.1 g dry tissue in 80% (v/v) ethanol overnight at 25 °C with occasional shaking, and then filtered after incubation. The filtrate was evaporated until complete dryness then dissolved in a known volume of distilled water. TSS was analyzed using the method of [84] by mixing 3 mL of freshly prepared anthrone reagent with 0.1 mL of ethanolic extracts in incubating the mixture for 10 min in a boiling water bath, cooling the tubes, and recording the intensity of the developed color at 625 nm. Sucrose content was determined by hydrolysis of 0.1 mL of ethanolic extract with 0.1 mL KOH (5.4 N) in a boiling water bath for 10 min. Subsequently, 3 mL of freshly prepared anthrone reagent were then mixed with the cooled reaction product, and the mixture was incubated for 5 min in a boiling water bath and cooled to room temperature. The intensity of the developed color was recorded at 620 nm [85].

TC was determined by anthrone method as described by [86]. Mixtures of 0.1 g of dry plant tissues and 5 mL of HCl (2.5 N) were kept in a boiling water bath for 3 h, then neutralized by sodium carbonate Na_2_CO_3_ after cooling to room temperature. Afterward, 1 mL of the extract was mixed with 4 mL of freshly prepared anthrone reagent in a boiling water bath for 8 min and cooled down; the intensity of the developed color was recorded at 630 nm.

Soluble protein extraction was carried out in Tris–HCl buffer according to the method adopted by Scarponi and Perucci [87]. Soluble protein was then determined spectrophotometrically by mixing 980 μL of protein reagent (Coomassie Brilliant Blue G250) with 20 μL of the extract and shaken vigorously. The contents of soluble protein were measured by monitoring the absorbance at 595 nm and a bovine serum albumin standard curve [88].

Proline was extracted in distilled water and determined following the method of [89]. A mixture of 2 mL of proline extract, 2 mL of acid ninhydrin, and 2 mL of glacial acetic acid was heated in a boiling water bath for an hour, then cooled in an ice bath. The absorbance of the developed color was recorded at 520 nm, and the concentration of proline was expressed as mg g^−1^ DW.

### 4.5. Determination of Electrolyte Leakage (EL) and Oxidative Damages

Electrolyte leakage was measured using freshly harvested plant leaves according to Shi et al. [90] using EC meter (HANNA Instrument, HI 8033). Leaves were cut into thin discs, transferred to test tube containing 30 mL of distilled deionized water, and incubated in the dark at room temperature for 24 h. The initial electric conductivity (EC1) was then recorded. After incubation, tubes were heated in a boiling water bath at 95 °C for 20 min and then cooled to room temperature. The final electrical conductivity (EC2) was determined and the El% was calculated using the equation: EL = (EC1/EC2) × 100.

Malondialdehyde concentration was measured using thiobarbituric acid (TBA) method [91]. Known weight of the frozen leaf tissue was homogenized in 0.1% trichloroacetic acid (TCA) at 4 ℃ and centrifugated at 12,000 rpm for 5 min. One ml of the clear supernatant was added to 4 mL of 20% TCA containing 0.5% TBA. The mixture was heated for 30 min at 90 °C then cooled and centrifugated again at 10,000 rpm for 5 min. The absorbance (A) was measured at 532 and 600 nm. MDA content was measured by subtracting the readings at A600 from those at A532 using an extension coefficient of 155 × 10^−3^ µM^−1^ cm^−1^, MDA concentration was expressed as µmol g^−1^ FW.

### 4.6. Non-Enzymatic Antioxidant Compounds

Total phenols and flavonoids were extracted by grinding 0.1 g powdered dry leave tissues in methanol according to Kosem et al. [92]. For total phenolics, 50 µL of the methanolic leaf extract was mixed with 400 µL of Folin–Ciocalteu reagent and incubated at 25 °C for 3 min. Subsequently, 800 µL of sodium carbonate (10%) were added, and the tubes were vortexed and kept in the dark at 25 °C for 2 h. The absorbance was measured at 765 nm using Shimadzu spectrophotometer (model UV-160A). The concentration of the total phenols content was expressed as mg of gallic acid equivalents (GAE) g^−1^ DW [93].

The total flavonoid content was determined using the spectrophotometric method [94]. Aliquots (1 mL) of the methanolic extracts were added to 4 mL of dist. H_2_O and 0.3 mL of NaNO_2_ solution (5%). After 5 min, 0.3 mL of 10% AlCl_3_ was added, and the tubes were incubated at 25 °C for 6 min. Afterward, 2 mL of 1 N NaOH were added, followed by addition of 2.4 mL of distilled water. The reaction mixture was mixed well, and the absorbance of the formed color was recorded at 510 nm using Shimadzu spectrophotometer (model UV-160A). Total flavonoid content was expressed as mg quercetin equivalent g^−1^ DW.

### 4.7. Antioxidant Enzymes Activities

Frozen fresh leaf samples were homogenized in 7 mL of cold 0.02 M phosphate buffer (pH 7) at 4 °C. The homogenates were centrifuged at 10,000 rpm for 20 min at 4 °C, and the supernatant (enzyme extract) was used for the measurements of antioxidant enzyme activities [95]. All measurements were carried out at 25 °C using Shimadzu spectrophotometer (model UV-160A). CAT activity was assayed by the method of [96]. Aliquots (0.5 mL) of enzyme extract were added to a mixture of 1 mL of phosphate buffer, pH 7.0 (0.01 M), 0.50 mL of H_2_O_2_ (0.20 M), and 0.40 mL of H_2_O. The enzymic reaction was incubated for one minute at 25 °C, then stopped by adding 2 mL of acid reagent (dichromate/acetic acid mixture). The enzyme mixture was heated for 10 min and cooled to 25 °C, then the absorbance was measured at 610 nm. Catalase activity was expressed in mM (H_2_O_2_ consumed) min^−1^ g^−1^ FW. Peroxidase was assayed by adding 0.1 mL of the enzyme extract to a mixture of 3 mL of 0.05 M pyrogallol prepared in 0.1 M phosphate buffer at pH 6 and 0.5 mL of 1% H_2_O_2_. The reaction was incubated for 1 min at 25 °C, and the reaction was stopped using 1 mL of 2.5 N H_2_SO_4_. The absorbance was measured at 420 nm. One enzyme unit is defined as unit min^−1^ g^−1^ FW [97].

### 4.8. Statistical Analysis

All the obtained data were analyzed by two-way analysis of variance (two-way ANOVA) using CoStat Version 6.3 software. Data are displayed as means ± SE. Post Hoc Duncan’s multiple range test was performed at *p* < 0.05. JMP Pro software was used to perform principal component analysis (PCA) and Pearson correlation.

## 5. Conclusions

Salinity stress caused significant retardation in the growth indices of the high yielding Giza 179 rice cultivar. Such inhibitory effect was associated with the disruption of mineral homeostasis, osmo-protection, and oxidative stress responses in Giza 179. Seed priming with humic acid significantly improved Giza 179 growth under non-saline conditions. It also effectively maintained the active growth of the salinity-stressed Giza 179 plants even at 8.00 mS/cm. Such humic acid ameliorating effects of the salt-induced damages were associated with ionic detoxification, oxidative stress alleviation, and osmotic regulation. Therefore, the current research provides new insights into both the potential of humic acid priming in the alleviation of the salinity-induced deleterious effects in Giza 179 plants and the underlaying physiological mechanisms for maintaining active growth under salinity stress. It also paves the road for more detailed field studies to explore the role of humic acid priming for enhancing growth and salinity tolerance of Giza 179 and possibly other rice cultivars in salt-affected lands.

## Figures and Tables

**Table 1:** Effect of seed priming with humic acid (100 mg/L) on growth parameters of Giza 179 plants grown under increased levels of seawater.

Parameters	Plant Length (cm)	Plant Fresh Weight (g)	Plant Dry Weight (g)	Leaf Area (cm^2^)
Amendment				
Control	94.8 ± 2.19 b	10.3 ± 0.890 b	3.44 ± 0.317 b	30.9 ± 0.660 b
Humic acid	103 ± 2.53 a	13.2 ± 0.948 a	4.62 ± 0.373 a	35.0 ± 1.161 a
*p*-value	***	***	***	***
Salinity				
0%	115 ± 1.38 a	17.4 ± 0.675 a	6.27 ± 0.280 a	39.6 ± 1.362 a
5%	105 ± 1.30 b	14.3 ± 0.668 b	4.79 ± 0.251 b	33.7 ± 0.702 b
10%	92.0 ± 1.20 c	9.58 ± 0.632 c	3.24 ± 0.281 c	30.2 ± 0.552 c
12.5%	83.2 ± 0.72 d	5.73 ± 0.577 d	1.84 ± 0.182 d	28.3 ± 0.614 d
*p*-value	***	***	***	***
Amend. × salinity				
Control	110 ± 0.58 b	15.8 ± 0.715 b	5.48 ± 0.266 b	35.1 ± 0.917 b
C + 5%	100 ± 0.38 d	12.7 ± 0.461 c	4.26 ± 0.180 c	31.9 ± 0.412 c
C + 10%	88.1 ± 0.53 f	8.33 ± 0.705 d	2.66 ± 0.248 d	29.1 ± 0.858 d
C + 12.5%	80.8 ± 0.23 h	4.20 ± 0.410 e	1.37 ± 0.121 e	27.6 ± 0.755 d
Humic	120 ± 0.69 a	18.9 ± 0.802 a	7.06 ± 0.246 a	44.0 ± 0.815 a
H + 5%	109 ± 0.59 c	15.9 ± 0.940 b	5.32 ± 0.381 b	35.6 ± 0.897 b
H + 10%	96.0 ± 0.89 e	10.8 ± 0.844 c	3.81 ± 0.311 c	31.4 ± 0.398 c
H + 12.5%	85.5 ± 0.59 g	7.25 ± 0.683 d	2.30 ± 0.219 d	29.0 ± 0.946 d
*p*-value	**	ns	ns	***

Values are the means ± SE of seven replicates. Different letters in each column indicate significant differences at *p* < 0.05. ** and *** indicate significant differences at *p* < 0.01 and *p* < 0.001, respectively. Ns indicates non-significant difference.

**Table 2:** Effect of seed priming with humic acid (100 mg/L) on ionic contents of shoots and roots of Giza 179 plants grown under increased levels of seawater.

Parameters	Shoot			Root		
	Na^+^	K^+^	K^+^/Na^+^ Ratio	Na^+^	K^+^	K^+^/Na^+^ Ratio
Amendment						
Control	0.543 ± 0.032 a	0.532 ± 0.009 a	1.034 ± 0.083 b	0.537 ± 0.024 b	0.202 ± 0.011 b	0.394 ± 0.038 b
Humic acid	0.468 ± 0.033 b	0.496 ± 0.009 b	1.151 ± 0.119 a	0.594 ± 0.025 a	0.251 ± 0.006 a	0.436 ± 0.029 a
*p*-value	***	***	**	***	***	***
Salinity						
0%	0.347 ± 0.024 d	0.553 ± 0.014 a	1.624 ± 0.094 a	0.454 ± 0.012 d	0.263 ± 0.006 a	0.581 ± 0.006 a
5%	0.476 ± 0.012 c	0.518 ± 0.003 b	1.092 ± 0.025 b	0.539 ± 0.020 c	0.237 ± 0.012 b	0.439 ± 0.009 b
10%	0.566 ± 0.016 b	0.505 ± 0.007 b	0.894 ± 0.015 c	0.607 ± 0.017 b	0.213 ± 0.013 c	0.350 ± 0.015 c
12.5%	0.633 ± 0.021 a	0.481 ± 0.014 c	0.760 ± 0.006 d	0.663 ± 0.017 a	0.193 ± 0.017 d	0.291 ± 0.022 d
*p*-value	***	***	***	***	***	***
Amend. × salinity						
Control	0.393 ± 0.009 e	0.579 ± 0.005 a	1.47 ± 0.048 b	0.431 ± 0.006 e	0.250 ± 0.002 bc	0.579 ± 0.002 a
C + 5%	0.502 ± 0.003 c	0.522 ± 0.002 b	1.04 ± 0.011 cd	0.508 ± 0.031 d	0.215 ± 0.012 d	0.423 ± 0.001 b
C + 10%	0.595 ± 0.018 b	0.518 ± 0.005 b	0.870 ± 0.019 e	0.574 ± 0.018 c	0.184 ± 0.005 e	0.322 ± 0.019 d
C + 12.5%	0.677 ± 0.009 a	0.509 ± 0.010 bc	0.751 ± 0.004 e	0.634 ± 0.022 b	0.158 ± 0.010 f	0.250 ± 0.024 e
Humic	0.300 ± 0.025 f	0.526 ± 0.015 b	1.77 ± 0.139 a	0.475 ± 0.012 de	0.276 ± 0.002 a	0.582 ± 0.013 a
H + 5%	0.448 ± 0.003 d	0.513 ± 0.002 bc	1.15 ± 0.013 c	0.568 ± 0.003 c	0.259 ± 0.007 ab	0.455 ± 0.010 b
H + 10%	0.535 ± 0.003 c	0.491 ± 0.005 c	0.917 ± 0.014 de	0.639 ± 0.006 b	0.241 ± 0.002 bc	0.377 ± 0.007 c
H + 12.5%	0.588 ± 0.009 b	0.452 ± 0.007 d	0.768 ± 0.010 e	0.691 ± 0.011 a	0.228 ± 0.005 cd	0.330 ± 0.011 d
*p*-value	ns	*	ns	ns	*	ns

Values are the means ± SE of three replicates. K^+^ and Na^+^ ions are expressed as mmol g^−1^ DW. Different letters in each column indicate significant differences at *p* < 0.05. *, **, and *** indicate significant differences at *p* < 0.05, *p* < 0.01, and *p* < 0.001, respectively. Ns indicates non-significant difference.

**Table 3:** Two-way ANOVA showing the effect of seed priming with humic acid (100 mg/L) on oxidative stress parameters, osmolytes content, and antioxidant activities of Giza 179 plants grown under increased levels of seawater.

Parameters	MDA	EL%	TSS	Sucrose	TC	Soluble Proteins	Proline	Flavonoids	Phenols	CAT	POX
Amendment											
Control	29.7 ± 0.943 a	59.1 ± 3.883 a	30.7 ± 1.061 a	8.02 ± 0.297 a	254 ± 5.783 b	8.29 ±0.487 b	0.679 ± 0.053 a	0.350 ± 0.010 a	1.08 ± 0.028 a	24.2 ± 1.817 b	11.0 ± 0.368 b
Humic acid	27.1 ± 0.808 b	47.6 ± 3.756 b	29.2 ± 0.580 b	7.26 ± 0.246 b	303 ± 5.580 a	10.3 ± 0.570 a	0.588 ± 0.020 b	0.332 ± 0.010 b	1.03 ± 0.034 b	30.5 ± 1.252 a	13.1 ± 0.558 a
*p*-value	***	***	***	***	***	***	***	***	***	***	***
Salinity											
0%	24.8 ± 0.480 d	35.5 ± 2.576 d	26.7 ± 0.307 d	6.41 ± 0.192 d	250 ± 11.671 c	6.90 ± 0.539 d	0.515 ± 0.005 d	0.301 ± 0.003 d	0.908 ± 0.023 d	21.2 ± 2.175 c	10.2 ± 0.346 c
5%	27.2 ± 0.753 c	50.9 ± 2.751 c	29.0 ± 0.201 c	7.48 ± 0.200 c	279 ± 11.981 b	8.58 ± 0.231 c	0.543 ± 0.005 c	0.320 ± 0.004 c	1.05 ± 0.009 c	27.1 ± 0.993 b	13.3 ± 0.969 a
10%	29.6 ± 0.965 b	56.8 ± 3.186 b	30.0 ± 0.443 b	7.86 ± 0.148 b	301 ± 10.350 a	10.5 ± 0.557 b	0.653 ± 0.026 b	0.358 ± 0.007 b	1.08 ± 0.007 b	26.4 ± 1.974 b	11.2 ± 0.288 b
12.5%	32.0 ± 0.770 a	70.1 ± 2.720 a	34.1 ± 0.866 a	8.82 ± 0.275 a	283 ± 11.365 b	11.3 ± 0.587 a	0.823 ± 0.058 a	0.386 ± 0.005 a	1.19 ± 0.012 a	34.8 ± 0.975 a	13.5 ± 0.327 a
*p*-value	***	***	***	***	***	***	***	***	***	***	***
Amend. × salinity (*p*-value)	ns	ns	***	ns	ns	***	***	ns	*	**	***

Values are the means ± SE of three replicates. Different letters in each column indicate significant differences at *p* < 0.05. *, **, and *** indicate significant difference at *p* < 0.05, *p* < 0.01, and *p* < 0.001, respectively. Ns indicates non-significant difference.

## Data Availability

Not applicable.

## References

[1] Lou W., Wu L., Chen H., Ji Z., Sun Y. (2012). Assessment of rice yield loss due to torrential rain: A case study of Yuhang County, Zhejiang Province, China. Nat. Hazards.

[2] Mishra B.K., Chaturvedi G.S. (2018). Flowering stage drought stress resistance in upland rice in relation to physiological, biochemical traits and yield. Int. J. Curr. Microbiol. Appl. Sci..

[3] Verma D.K., Srivastav P.P. (2020). Bioactive compounds of rice (*Oryza sativa* L.): Review on paradigm and its potential benefit in human health. Trends Food Sci. Technol..

[4] Das P., Nutan K.K., Singla-Pareek S.L., Pareek A. (2015). Understanding salinity responses and adopting ‘omics-based’ approaches to generate salinity tolerant cultivars of rice. Front. Plant Sci..

[5] Hossain M.S. (2019). Present scenario of global salt affected soils, its management and importance of salinity research. Int. Res. J. Biol. Sci..

[6] Amirjani M.R. (2011). Effect of salinity stress on growth, sugar content, pigments and enzyme activity of rice. Int. J. Bot..

[7] Munns R., Tester M. (2008). Mechanisms of salinity tolerance. Ann. Rev. Plant Biol..

[8] Hasanuzzaman M., Nahar K., Fujita M., Ahmad P., Azooz M.M., Prasad M.N.V. (2013). Plant response to salt stress and role of exogenous protectants to mitigate salt-induced damages. Ecophysiology and Responses of Plants under Salt Stress.

[9] Rahman A., Nahar K., AL Mahmud J., Hasanuzzaman M., Hossain M.S., Fujita M., Li J. (2017). Salt stress tolerance in rice: Emerging role of exogenous phytoprotectants. Advances in International Rice Research.

[10] Hasanuzzaman M., Raihan M.R.H., Masud A.A.C., Rahman K., Nowroz F., Rahman M., Nahar K., Fujita M. (2021). Regulation of reactive oxygen species and antioxidant defense in plants under salinity. Int. J. Mol. Sci..

[11] Kumar P., Kumar T., Singh S., Tuteja N., Prasad R., Singh J. (2020). Potassium: A key modulator for cell homeostasis. J. Biotechnol..

[12] Horie T., Karahara I., Katsuhara M. (2012). Salinity tolerance mechanisms in glycophytes: An overview with the central focus on rice plants. Rice.

[13] Ibrahim E.A. (2016). Seed priming to alleviate salinity stress in germinating seeds. J. Plant Physiol..

[14] Çimrin K.M., Türkmen Ö., Turan M., Tuncer B. (2010). Phosphorus and humic acid application alleviate salinity stress of pepper seedling. Afr. J. Biotechnol..

[15] Canellas L.P., Canellas N.O.A., Irineu L.E.S.D.S., Olivares F.L., Piccolo A. (2020). Plant chemical priming by humic acids. Chem. Biol. Technol. Agric..

[16] Yakhin O.I., Lubyanov A.A., Yakhin I.A., Brown P.H. (2017). Biostimulants in plant science: A global perspective. Front. Plant Sci..

[17] Goel P., Dhingra M., Makan A. (2021). Humic substances: Prospects for use in agriculture and medicine. Humic Substances.

[18] García A.C., Santos L.A., Izquierdo F.G., Sperandio M.V.L., Castro R.N., Berbara R.L.L. (2012). Vermicompost humic acids as an ecological pathway to protect rice plant against oxidative stress. Ecol. Eng..

[19] Elmoghazy A.M., Elshenawy M.M., Negm A.M., Abu-hashim M. (2018). Sustainable cultivation of rice in Egypt. Sustainability of Agricultural Environment in Egypt: Part I.

[20] Yang Y., Guo Y. (2018). Unraveling salt stress signaling in plants. J. Integr. Plant Biol..

[21] Shahid M.A., Sarkhosh A., Khan N., Balal R.M., Ali S., Rossi L., Gómez C., Mattson N., Nasim W., Garcia-Sanchez F. (2020). Insights into the physiological and biochemical impacts of salt stress on plant growth and development. Agronomy.

[22] Abdallah M.M.S., Abdelgawad Z.A., EL-Bassiouny H.M.S. (2016). Alleviation of the adverse effects of salinity stress using trehalose in two rice varieties. S. Afr. J. Bot..

[23] Singh V., Singh A.P., Bhadoria J., Giri J., Singh J., Vineeth T.V., Sharma P.C. (2018). Differential expression of salt-responsive genes to salinity stress in salt-tolerant and salt-sensitive rice (*Oryza sativa* L.) at seedling stage. Protoplasma.

[24] Rahman A., Nahar K., Hasanuzzaman M., Fujita M. (2016). Calcium supplementation improves Na^+^/K^+^ ratio, antioxidant defense and glyoxalase systems in salt-stressed rice seedlings. Front. Plant Sci..

[25] Kordrostami M., Rabiei B., Kumleh H.H. (2017). Biochemical, physiological and molecular evaluation of rice cultivars differing in salt tolerance at the seedling stage. Physiol. Mol. Biol. Plants.

[26] De Castro T.A.V.T., Berbara R.L.L., Tavares O.C.H., da Graça Mello D.F., Pereira E.G., de Souza C.D.C.B., Espinosa L.M., García A.C. (2021). Humic acids induce a eustress state via photosynthesis and nitrogen metabolism leading to a root growth improvement in rice plants. Plant Physiol. Biochem..

[27] Tavares O.C.H., Santos L.A., Filho D.F., Ferreira L.M., Garcia A.C., Castro T.A.V.T., Zonta E., Pereira M.G., Fernandes M.S. (2021). Response surface modeling of humic acid stimulation of the rice (*Oryza sativa* L.) root system. Arch. Agron. Soil Sci..

[28] Matuszak-Slamani R., Bejger R., Cieśla J., Bieganowski A., Koczańska M., Gawlik A., Kulpa D., Sienkiewicz M., Włodarczyk M., Gołębiowska D. (2017). Influence of humic acid molecular fractions on growth and development of soybean seedlings under salt stress. Plant Growth Regul..

[29] Kaya C., Akram N.A., Ashraf M., Sonmez O. (2018). Exogenous application of humic acid mitigates salinity stress in maize (*Zea mays* L.) plants by improving some key physico-biochemical attributes. Cereal Res. Commun..

[30] Ouni Y., Ghnaya T., Montemurro F., Abdelly C., Lakhdar A. (2014). The role of humic substances in mitigating the harmful effects of soil salinity and improve plant productivity. Int. J. Plant Prod..

[31] Trevisan S., Francioso O., Quaggiotti S., Nardi S. (2010). Humic substances biological activity at the plant-soil interface: From environmental aspects to molecular factors. Plant Signal. Behav..

[32] Taha S.S., Osman A.S. (2018). Influence of potassium humate on biochemical and agronomic attributes of bean plants grown on saline soil. J. Horticult. Sci. Biotechnol..

[33] Ali A.Y.A., Ibrahim M.E.H., Zhou G., Zhu G., Elsiddig A.M.I., Suliman M.S.E., Elradi S.B.M., Salah E.G.I. (2022). Interactive impacts of soil salinity and jasmonic acid and humic acid on growth parameters, forage yield and photosynthesis parameters of sorghum plants. S. Afr. J. Bot..

[34] Sisodia A., Padhi M., Pal A.K., Barman K., Singh A.K., Rakshit A., Singh H.B. (2018). Seed priming on germination, growth and flowering in flowers and ornamental trees. Advances in Seed Priming.

[35] García A.C., Van Tol De Castro T.A., Santos L.A., Tavares O.C.H., Castro R.N., Berbara R.L.L., García-Mina J.M. (2019). Structure–property–function relationship of humic substances in modulating the root growth of plants: A review. J. Environ. Qual..

[36] Olk D.C., Bloom P.R., Perdue E.M., McKnight D.M., Chen Y., Farenhorst A., Senesi N., Chin Y.P., Schmitt-Kopplin P., Hertkorn N. (2019). Environmental and agricultural relevance of humic fractions extracted by alkali from soils and natural waters. J. Environ. Qual..

[37] Ketehouli T., Carther K.F.I., Noman M., Wang F.W., Li X.W., Li H.Y. (2019). Adaptation of plants to salt stress: Characterization of Na^+^ and K^+^ transporters and role of CBL gene family in regulating salt stress response. Agronomy.

[38] Ibrahimova U., Kumari P., Yadav S., Rastogi A., Antala M., Suleymanova Z., Zivcak M., Tahjib-Ul-Arif M., Hussain S., Abdelhamid M. (2021). Progress in understanding salt stress response in plants using biotechnological tools. J. Biotechnol..

[39] Azarin K.V., Alabushev A.V., Usatov A.V., Kostylev P.I., Kolokolova N.S., Usatova O.A. (2016). Effects of salt stress on ion balance at vegetative stage in rice (*Oryza sativa* L.). Online J. Biol. Sci..

[40] Porcel R., Aroca R., Azcon R., Ruiz-Lozano J.M. (2016). Regulation of cation transporter genes by the arbuscular mycorrhizal symbiosis in rice plants subjected to salinity suggests improved salt tolerance due to reduced Na^+^ root-to-shoot distribution. Mycorrhiza.

[41] Yan G., Fan X., Peng M., Yin C., Xiao Z., Liang Y. (2020). Silicon improves rice salinity resistance by alleviating ionic toxicity and osmotic constraint in an organ-specific pattern. Front. Plant Sci..

[42] Ueda A., Yahagi H., Fujikawa Y., Nagaoka T., Esaka M., Calcaño M., González M.M., Martich J.D.H., Saneoka H. (2013). Comparative physiological analysis of salinity tolerance in rice. Soil Sci. Plant Nutr..

[43] Chakraborty K., Basak N., Bhaduri D., Ray S., Vijayan J., Chattopadhyay K., Sarkar R.K., Hasanuzzaman M., Fujita M., Oku H., Nahar K., Hawrylak-Nowak B. (2018). Ionic basis of salt tolerance in plants: Nutrient homeostasis and oxidative stress tolerance. Plant Nutrients and Abiotic Stress Tolerance.

[44] Khaleda L., Park H.J., Yun D.J., Jeon J.R., Kim M.G., Cha J.Y., Kim W.Y. (2017). Humic acid confers high-affinity K^+^ transporter 1-mediated salinity stress tolerance in Arabidopsis. Mol. Cells.

[45] Ghosh N., Das S.P., Mandal C., Gupta S., Das K., Dey N., Adak M.K. (2012). Variations of antioxidative responses in two rice cultivars with polyamine treatment under salinity stress. Physiol. Mol. Biol. Plants.

[46] Cai W., Liu W., Wang W.S., Fu Z.W., Han T.T., Lu Y.T. (2015). Overexpression of rat neurons nitric oxide synthase in rice enhances drought and salt tolerance. PLoS ONE.

[47] Xu L., Wang A., Wang J., Wei Q., Zhang W. (2017). *Piriformospora indica* confers drought tolerance on *Zea mays* L. through enhancement of antioxidant activity and expression of drought-related genes. Crop. J..

[48] Tahjib-Ul-Arif M., Roy P.R., Sohag A.A.M., Afrin S., Rady M.M., Hossain M.A. (2018). Exogenous calcium supplementation improves salinity tolerance in BRRI dhan28; a salt-susceptible high-yielding *Oryza sativa* cultivar. J. Crop. Sci. Biotechnol..

[49] Shultana R., Zuan A.T.K., Yusop M.R., Saud H.M., Ayanda A.F. (2020). Effect of salt-tolerant bacterial inoculations on rice seedlings differing in salt-tolerance under saline soil conditions. Agronomy.

[50] Hatami E., Shokouhian A.A., Ghanbari A.R., Naseri L.A. (2018). Alleviating salt stress in almond rootstocks using of humic acid. Sci. Hortic..

[51] Bano S., Ahmed M.Z., Abideen Z., Qasim M., Gul B., Khan N.U. (2022). Humic acid overcomes salinity barriers and stimulates growth of *Urochondra setulosa* by altering ion-flux and photochemistry. Acta Physiol. Plant..

[52] Chen H., Jiang J.G. (2010). Osmotic adjustment and plant adaptation to environmental changes related to drought and salinity. Environ. Rev..

[53] Abdelrahman M., Burritt D.J., Tran L.P. (2018). The use of metabolomic quantitative trait locus mapping and osmotic adjustment traits for the improvement of crop yields under environmental stresses. Semin. Cell Dev. Biol..

[54] Pattanagul W., Thitisaksakul M. (2008). Effect of salinity stress on growth and carbohydrate metabolism in three rice (*Oryza sativa* L.) cultivars differing in salinity tolerance. Indian J. Exp. Biol..

[55] Mushtaq Z., Faizan S., Gulzar B. (2020). Salt stress, its impacts on plants and the strategies plants are employing against it: A review. J. Appl. Biol. Biotechnol..

[56] Zahedi S.M., Hosseini M.S., Hoveizeh N.F., Gholami R., Abdelrahman M., Tran L.S.P. (2021). Exogenous melatonin mitigates salinity-induced damage in olive seedlings by modulating ion homeostasis, antioxidant defense, and phytohormone balance. Physiol. Plant..

[57] Mishra N., Srivastava A.P., Esmaeili N., Hu W., Shen G. (2018). Overexpression of the rice gene OsSIZ1 in Arabidopsis improves drought-, heat-, and salt-tolerance simultaneously. PLoS ONE.

[58] Khanna-Chopra R., Semwal V.K., Lakra N., Pareek A., Hasanuzzaman M., Fujita M., Oku H., Islam M.T. (2019). Proline—A key regulator conferring plant tolerance to salinity and drought. Plant Tolerance to Environmental Stress: Role of Phytoprotectants.

[59] Kosová K., Vítámvás P., Prášil I.T., Renaut J. (2011). Plant proteome changes under abiotic stress—Contribution of proteomics studies to understanding plant stress response. J. Proteom..

[60] Thitisaksakul M., Arias M.C., Dong S., Beckles D.M. (2017). Overexpression of GSK3-like Kinase 5 (OsGSK5) in rice (*Oryza sativa*) enhances salinity tolerance in part via preferential carbon allocation to root starch. Funct. Plant Biol..

[61] Dong S., Beckles D.M. (2019). Dynamic changes in the starch-sugar interconversion within plant source and sink tissues promote a better abiotic stress response. J. Plant Physiol..

[62] Jarošová M., Klejdus B., Kováčik J., Babula P., Hedbavny J. (2016). Humic acid protects barley against salinity. Acta Physiol. Plant..

[63] Yildiztekin M., Tuna A.L., Kaya C. (2018). Physiological effects of the brown seaweed (*Ascophyllum nodosum*) and humic substances on plant growth, enzyme activities of certain pepper plants grown under salt stress. Acta Biol. Hung..

[64] Mazhar A.A.M., Shedeed S.I., Abdel-Aziz N.G., Mahgoub M.H. (2012). Growth, flowering and chemical constituents of *Chrysanthemum indicum* L. plant in response to different levels of humic acid and salinity. J. Appl. Sci. Res..

[65] Roomi S., Masi A., Conselvan G.B., Trevisan S., Quaggiotti S., Pivato M., Arrigoni G., Yasmin T., Carletti P. (2018). Protein profiling of Arabidopsis roots treated with humic substances: Insights into the metabolic and interactome networks. Front. Plant Sci..

[66] Ahmad P. (2014). Oxidative Damage to Plants: Antioxidant Networks and Signaling.

[67] Zhao C., Zhang H., Song C., Zhu J.K., Shabala S. (2020). Mechanisms of plant responses and adaptation to soil salinity. Innovation.

[68] Ahmad R., Hussain S., Anjum M.A., Khalid M.F., Saqib M., Zakir I., Hassan A., Fahad S., Ahmad S., Hasanuzzaman M., Hakeem K.R., Nahar K., Alharby H.F. (2019). Oxidative stress and antioxidant defense mechanisms in plants under salt stress. Plant Abiotic Stress Tolerance.

[69] Sheteiwy M.S., An J., Yin M., Jia X., Guan Y., He F., Hu J. (2019). Cold plasma treatment and exogenous salicylic acid priming enhances salinity tolerance of *Oryza sativa* seedlings. Protoplasma.

[70] Khan M.A., Hamayun M., Asaf S., Khan M., Yun B.W., Kang S.M., Lee I.J. (2021). *Rhizospheric Bacillus* spp. rescues plant growth under salinity stress via regulating gene expression, endogenous hormones, and antioxidant system of *Oryza sativa* L. Front. Plant Sci..

[71] Hasanuzzaman M., Alam M.M., Rahman A., Hasanuzzaman M., Nahar K., Fujita M. (2014). Exogenous proline and glycine betaine mediated upregulation of antioxidant defense and glyoxalase systems provides better protection against salt-induced oxidative stress in two rice (*Oryza sativa* L.) varieties. Biomed Res. Int..

[72] Minh L.T., Khang D.T., Ha P.T.T., Tuyen P.T., Minh T.N., Quan N.V., Xuan T.D. (2016). Effects of salinity stress on growth and phenolics of rice (*Oryza sativa* L.). Int. Lett. Nat. Sci..

[73] Liu G., Zhu W., Zhang J., Song D., Zhuang L., Ma Q., Yang X., Liu X., Zhang J., Zhang H. (2022). Antioxidant capacity of phenolic compounds separated from tea seed oil in vitro and in vivo. Food Chem..

[74] Ben Abdallah S., Aung B., Amyot L., Lalin I., Lachâal M., Karray-Bouraoui N., Hannoufa A. (2016). Salt stress (NaCl) affects plant growth and branch pathways of carotenoid and flavonoid biosyntheses in *Solanum nigrum*. Acta Physiol. Plant..

[75] García A.C., Santos L.A., de Souza L.G.A., Tavares O.C.H., Zonta E., Gomes E.T.M., García-Mina J.M., Berbara R.L.L. (2016). Vermicompost humic acids modulate the accumulation and metabolism of ROS in rice plants. J. Plant Physiol..

[76] Cordeiro F.C., Santa-Catarina C., Silveira V., de Souza S.R. (2011). Humic acid effect on catalase activity and the generation of reactive oxygen species in corn (*Zea mays*). Biosci. Biotechnol. Biochem..

[77] Mansoor S., Ali Wani O., Lone J.K., Manhas S., Kour N., Alam P., Ahmad A., Ahmad P. (2022). Reactive Oxygen Species in plants: From source to sink. Antioxidants.

[78] Abbas G., Rehman S., Siddiqui M.H., Ali H.M., Farooq M.A., Chen Y. (2022). Potassium and humic acid synergistically increase salt tolerance and nutrient uptake in contrasting wheat genotypes through ionic homeostasis and activation of antioxidant enzymes. Plants.

[79] Ashour H.A., Esmail S.E.A., Kotb M.S. (2021). Alleviative effects of chitosan or humic acid on *Vitex trifolia* ‘Purpurea’ grown under salinity stress. Ornam. Horticult..

[80] Mridha D., Paul I., De A., Ray I., Das A., Joardar M., Chowdhury N.R., Bhadoria P.B.S., Roychowdhury T. (2021). Rice seed (IR64) priming with potassium humate for improvement of seed germination, seedling growth and antioxidant defense system under arsenic stress. Ecotoxicol. Environ. Saf..

[81] Treutter D. (2006). Significance of flavonoids in plant resistance: A review. Environ. Chem. Lett..

[82] Palaniswamy K.M., Gomez K.A. (1974). Length-width method for estimating leaf area of rice. Agron. J..

[83] Motsara M.R., Roy R.N. (2008). Guide to Laboratory Establishment for Plant Nutrient Analysis.

[84] Yemm E.W., Willis A.J. (1954). The estimation of carbohydrates in plant extracts by anthrone. Biochem. J..

[85] Van Handel E. (1968). Direct microdetermination of sucrose. Anal. Biochem..

[86] Hedge J.E., Hofreiter B.T., Whistler R.L., BeMiller J.N. (1962). Methods in Carbohydrate Chemistry.

[87] Scarponi L., Perucci P. (1986). The effect of a number of S-triazines on the activity of maize delta aminolivulinate dehydratase. Agrochimica.

[88] Bradford M.M. (1976). A rapid and sensitive method for the quantitation of microgram quantities of protein utilizing the principle of protein-dye binding. Anal. Biochem..

[89] Bates L.S., Waldren R.P., Teare I.D. (1973). Rapid determination of free proline for water-stress studies. Plant Soil.

[90] Shi Q., Bao Z., Zhu Z., Ying Q., Qian Q. (2006). Effects of different treatments of salicylic acid on heat tolerance, chlorophyll fluorescence, and antioxidant enzyme activity in seedlings of *Cucumis sativa* L. Plant Growth Regul..

[91] Heath R.L., Packer L. (1968). Photoperoxidation in isolated chloroplasts: I. Kinetics and stoichiometry of fatty acid peroxidation. Arch. Biochem. Biophys..

[92] Kosem N., Han Y.H., Moongkarndi P. (2007). Antioxidant and cytoprotective activities of methanolic extract from *Garcinia mangostana* hulls. Scienceasia.

[93] Singleton V.L., Rossi J.A. (1965). Colorimetry of total phenolics with phosphomolybdic-phosphotungstic acid reagents. Am. J. Enol. Vitic..

[94] Marinova D., Ribarova F., Atanassova M. (2005). Total phenolics and total flavonoids in bulgarian fruits and vegetables. J. Univ. Chem. Technol. Metall..

[95] Agarwal S., Shaheen R. (2007). Stimulation of antioxidant system and lipid peroxidation by abiotic stresses in leaves of *Momordica charantia*. Braz. J. Plant Physiol..

[96] Sinha A.K. (1972). Colorimetric assay of catalase. Anal. Biochem..

[97] Devi P. (2002). Principles and Methods in Plant Molecular Biology, Biochemistry and Genetics.

